# Isolation of nucleic acids using silicon dioxide powder as a tool for environmental monitoring

**DOI:** 10.1007/s10661-019-7840-2

**Published:** 2019-11-08

**Authors:** Jacek Urbaniak, Daniel Janowski, Brayan Jacewski

**Affiliations:** 1 Department of Botany and Plant Ecology, Wrocław University of Plant Sciences, pl. Grunwaldzki 24a, 50-363 Wrocław, Poland; 2 Institute of Dendrobiology PAS, ul. Parkowa 5, 62-035 Kórnik, Poland

**Keywords:** Species, Fungi, DNA, Method, Nucleic acids, Purification, Silicon dioxide

## Abstract

Isolation and purification of nucleic acids are basic laboratory procedures used in molecular analysis supporting determination of organisms in environmental monitoring. However, many different methods of isolation are commonly used, often being designed for a particular type of DNA extraction. While researchers commonly decide on commercial isolation kits for their ease of use and efficiency, they require large amounts of studied tissue, and the cost of purchasing such kits over a long run can be high. To provide an alternative to using commercial kits, we have developed a simple, rapid, cost-effective, and reliable protocol for DNA isolation from cultured fungi on slants and from dried fungal samples using silica particles (silicon dioxide powder) in chaotropic conditions. With the presented method, it is possible to isolate good-quality DNA from fungi in less than 1.5 h, using easily accessible chemicals. Compared with other methods employing CTAB or commercial kits, it allows fast, easy, and cheap DNA purification from two main sources of fungi routinely used for research. In addition to the method protocol, we also provide advice for further optimization of the isolation process to account for specific conditions, making the procedure more useful.

## Introduction

In different areas of environmental monitoring, when working with genera and species, there is still a risk of incorrect determination of the organisms and to provide monitoring of invasive or potentially risky species (Pusz et al. 2017). Numerous methods based on morphological features or on biochemical reactions usually allow for correct determination. Unfortunately, morphological criteria are sometimes not well met by traditional approaches, including identification based on organism morphology (Darling and Blum 2007), and are insufficient in mycology, especially when microscopic fungi need to be determined. Then, molecular methods based on gene sequencing are really helpful. However, a fast and easy determination based on DNA tools has one critical step: the nucleic acid extraction, which has to be done fast, easily, and cheaply. The extraction, from different materials like plants, fungi, or animal tissue, is a routinely performed method in every type of molecular analysis and research area: phylogenetic analysis, fingerprinting, or molecular cloning. Although numerous commercial methods based on the use of silica columns or magnetic beads for nucleic acid purification have been developed, other methods, such as numerous variants of the CTAB extraction, are widespread and still used (Cheng et al. 2003; Werth et al. 2016). Commercial kits are often disadvantageous, being designed only for a specific type of material (animal tissue, plants, blood, bacteria, or fungi), not to mention their relatively high costs which often limit their use. On the other hand, classical methods like CTAB extraction, CsCl gradient centrifugation, or phenol–chloroform extraction require toxic and irritant chemicals, are time-consuming, and may need expensive ultracentrifuges (Doyle and Doyle 1990; Murray and Thomson 1980).

In this paper, we would like to present a developed and optimized simple DNA purification method for fungi, using silica powder (Boom et al. 1990) and later adapted for other research (Li et al. 2010; Malinowski 1997; Zacharzewska et al. 2014)*.* In general, the method based on silica particles relies on nucleic acids binding to the silica surface in the presence of chaotropic salts, like guanidinium thiocyanate (GuSCN) or guanidinium hydrochloride (GuHCl)(Boom et al. 1990; Li et al. 2010). This approach proves to be versatile because it was used to purify plasmid DNA from *E. coli*, for detection of the potato virus Y or in isolating DNA from blood consumed by mosquitoes (Guelbéogo et al. 2018; Li et al. 2010; Zacharzewska et al. 2014). In contrast to highly optimized and efficient but expensive commercial kits, the silica-based method can be easily adapted to individual sub-protocols. It is sensitive, reproducible, rapid, and cheap and does not require expensive devices. It is suitable for routine use in environmental monitoring, and the quality of the final products is sufficient for them to be used in downstream applications, like PCR or nucleotide sequencing (Boom et al. 1990).

## Material and methods

### Preparation of fungal material

We have used tests for two types of fungal material: fungi cultured on agar slants and dried fungal samples (Table 1). In the case of the agar-cultured samples, first, small fungal samples were immersed in a disinfectant. Later, the samples were placed on a standard potato dextrose agar (PDA) medium in Petri dishes. After the growth of the fungi, they were passaged on agar slants, incubated at room temperature and used in this study. In the case of macroscopic fungi, they were collected in the wild, dried, and used in the study. Prior to the DNA isolation, all fungal samples were placed in the Retsch MM 400 Mixer Mill for 1.5 min at 20–30 Hz and homogenized.

**Table 1:** A list of fungi species used in this study

No.	Species name
Fungi from slants	
1s, 2s	*Alternaria alternata* (Fr.) Keissler 1912: 433
3s	*Cladosporium allicinum* (Fr. : Fr.) Bensch 2012: 72 (50)
4s	*Epicoccum nigrum* Link (1816): 8 (32)
5s, 6s	*Alternaria infectoria* E.G. Simmons (1986): 25 (1)
Macroscopic fungi	
7b	*Xerocomus badius* (Fr.) E.-J. Gilbert (1931): 3(92)
8b	*Boletus chrysenteron* Bull. (1791): 490 (3)
9b	*Boletus edulis* Bull. (1782): 2 (60)
10b	*Boletus reticulatus* (Hoffm.) Pers (1801): 548

### DNA isolation methods

The nucleic acids from the macroscopic fungi and from slants were isolated using three different methods: commercial kits, CTAB method (Doyle and Doyle 1987), and using silica powder (Table 2)*.*

**Table 2:** Cost and efficiency of DNA isolation methods

Isolation method/manufacturer	NucleoSpin® Plant II/Macherey Nagel	DNeasy Plant Mini Kit/Qiagen	CTAB	Silica
Cost of isolation [EUR]	3	3.5	1.3	0.9
Estimated purification time—8 samples [h]	3	3	5	1.5
Concentration min–max (median) [μg/μl]				
Agar slants	27–35 (29)	29–39 (34)	36–131 (124)	6–56 (17)
Macroscopic fungi	29–46 (48)	38–78 (54)	97–110 (102)	35–80 (56)
Quality (260/280 ratio)				
Agar slants	1.5–1.6	1.4–1.5	×–1.7	1.2–1.9
Macroscopic fungi	1.4–1.5	1.6–1.6	×–1.8	1.3–1.8

Min–max, median value is given in brackets

^a^In case of the Macherey Nagel and Qiagen Kits, we used a set for 250 isolations

Following the user manual procedure provided by the producer, we have used the NucleoSpin® Plant II DNA Kit (Macherey Nagel, Düren, Germany) and DNeasy® Plant Mini Kit (Qiagen, Hilden, Germany). We have also used the CTAB method by Doyle and Doyle (1987) adapted to isolated nucleic acids from fungi.

For the silicon dioxide–based isolation, silica suspension, as well as buffers L2 and L6, was prepared according to the recipe (Boom et al. 1990) with some modifications (Zacharzewska et al. 2014). For the silica suspension, silica powder (Honeywell/Fluka S5631) was used. After preparation of the silicon dioxide suspension and both buffers, we followed the protocol described below.

The protocol of fungal DNA isolation SiO2" in the same font style (bigger and bold) as another one below "Additional notes on the protocol_2_

1. Shortly prior to the isolation procedure, fill Eppendorf tubes with approx. 20 mg of fungal material.
2. Add stainless steel–coated metal balls, one–two per tube, and grind by shaking in a ball mill set (we used Mixer Mill MM400; Retsch, Haan, Germany, 20–30 Hz for 1–1.5 min).
3. Add 900 μl of L6 buffer and 40 μl of the previously prepared silica suspension to the homogenized samples, vortex and briefly centrifuge (2 min, 12,000 rcf/room temperature).
4. Transfer 50 μl of the supernatant from each of the samples to newly prepared tubes, incubate in a thermoblock (10 min, 1000 rcf, room temperature), and centrifuge (15 min, 12,000 rcf/room temperature).
5. Remove supernatant from the tubes and wash the remaining pellet: twice with 1 ml of L2 buffer, twice with 1 ml of 70% ethanol, and once with 1 ml of acetone, by resuspending the pellet in the wash medium; briefly centrifuge (2 min, 12,000 rcf/room temperature). Remove supernatant.
6. Dry the remaining pellets at room temperature or in a vacuum centrifuge at low pressure (we used SpeedVac Concentrator, 47 °C, Savant DNA110, Thermo, Waltham, MA, USA).
7. Resuspend the dry pellet in 30–50 μl of 0.05 M TE buffer, place in a thermoblock (10 min, 1000 rpm, 56 °C) and centrifuge (2 min, 12,000 rcf/room temperature). Move the supernatant into new tubes and store in a freezer for further use.

### Additional notes on the protocol

1. Guanidinium thiocyanate (GuSCN) is a more effective agent in the purification of DNA than guanidinium hydrochloride (GuHCl)(Boom et al. 1990). The low pH and presence of chaotropic salts not only facilitate the DNA isolation but also serve an additional purpose, by lysing cells and protecting DNA from degradation by denaturing nucleases (Boom et al. 1990; Brown 2016*;* Vandeventer et al. 2012). As an additional experiment, we have also performed isolations, where instead of the L6 buffer, different lysis buffers taken directly from the commercial kits were used: (i) NucleoSpin® Plant II DNA Kit—the PL1 lysis buffer based on established CTAB method containing cetyltrimethylammonium bromide, a cationic detergent; (ii) PL2 (NucleoSpin® Plant II DNA Kit) buffer that contains sodium dodecylsulfate—SDS;(iii) DNeasy® Plant Mini Kit—the AP1 buffer also with sodium dodecyl sulfate (SDS). Out of the three, the CTAB-based PL1 buffer provided the best results; however, the differences in yielded DNA were minuscule. Regardless of the buffer used, the product was successfully used as a DNA matrix for PCR reaction.
2. In an additional experiment, the applicability of our method for vascular plants was verified. We used cultivated Petroselinum crispum and dried high mountain plant Swertia perennis as test specimens. One hundred milligrams fresh material of P. crispum was mechanically disrupted using Mixer Mill (30 Hz for 2 min); centrifuged and freshly collected supernatant was used as a substrate to DNA isolation. In the case of S. perennis, we used material dried in silica gel orange. About 30 mg of leaves was mechanically disrupted and powdered using Mixer Mill (30 Hz for 2 min). The powdered samples were used for DNA isolation using silicon dioxide. In both cases, the DNA was isolated and amplified without problems, showing that the presented protocol works well also with higher plants.
3. We have used the described protocol for successful isolation of the DNA directly from the PCR amplification products. Cut bands (sliced gel agarose) from the agarose gel were placed in a PCR reaction tube, frozen at − 20 °C for 20 min, and centrifuged after placing in 1.5-ml Eppendorf tubes (10 min, 12,000 rcf/room temperature). The resulting solution was used instead of rough material in the isolation according to the described protocol. Successful isolation was later confirmed by separation on the agarose gel (visible bands were noted).
4. We have also tested replacing the mechanical disruption with freezing in liquid nitrogen, paired with manual disrupting in a mortar. We tested this option with all types of material, fresh and dried fungal and plant. This method also gave good results, confirmed with visible DNA bands on the agarose gel.
5. To check if the reagents used in our procedure may negatively affect the sensitivity of the PCR reaction, for a number of prepared DNA isolates, 1- and 10-fold dilutions were prepared and used as matrices for PCR amplification. We did not find any differences in 1- or 10-fold dilutions, as was confirmed by separation on the agarose gel. Regardless of the dilution, good-quality unfragmented product of a similar concentration was present.
6. We did not use the RNase for the RNA degradation; however, it can be used optionally, in step 3.
7. In step 7, optionally, different incubation time may be applied. We tested 5, 10, and 15 min of incubation, however, without visible differences in the DNA recovery rate.
8. The commonly used buffers for elution and digestion of nucleic acids present in commercial kits are composed of 10 mM Tris-HCl and 1 mM EDTA, pH 8.0 or 5 mM Tris-HCl, pH 8.5. We have used the 10 mM Tris-HCl, 1 mM EDTA, pH 8.0 *(*Boom et al. 1990); however, other elution buffers, as well as DNase-free sterile water (37 °C), may be used.

### Determination of DNA quality and quantity

The isolates were preliminarily assessed through electrophoresis on 1% agarose gel stained with ethidium bromide, with DNA molecular weight ladder as a reference point, as well as by spectrophotometric analysis using Eppendorf UV/VIS BioPhotometer (Eppendorf, Hamburg, Germany). The ratio A260/A280 was used to assess the purity of the isolated DNA and yield.

### PCR reactions

We have also performed the PCR reactions to confirm the quality of the product of silicon dioxide–based isolation. For this, the internal transcribed spacer region (ITS) was amplified using standard ITS-1F and ITS-4 primers (Pusz and Urbaniak 2017). PCR amplification products were again evaluated by electrophoresis using 100 bp DNA ladder (Thermo Fisher Scientific, Waltham, MA, USA) as a reference. PCR reaction mix (20 μl) contained the polymerase (1 U Taq recombinant polymerase, Thermo Fisher Scientific) with 1 mM MgCl_2_, 0.5 μM of each primer, 0.4 mM dNTP, and 1 μl DNA template. PCR was performed with a Veriti Thermal Cycler (Life Technologies, Carlsbad, CA, USA) set to the following parameters: 10 min at 95 °C, followed by 32 cycles; 45 s at 95 °C, 30 s at 49.2 °C, and 45 s at 72 °C, followed by a final extension step of 10 min at 72 °C. Prior to sequencing, PCR products were purified using Gene MATRIX PCR/DNA Clean Up Purification Kit (EURx, Gdańsk, Poland). Sequencing, post-reaction purification, and reading were done by Genomed (Warsaw, Poland) using an ABI 377XL Automated DNA Sequencer (Applied Biosystems, Carlsbad, CA, USA).

## Results

### Isolation and amplification results

Both commercial kits used for comparison yielded 27 to 35 (NucleoSpin) and 29 to 39 (DNeasy)μg/μl of total DNA in the case of isolating DNA of cultured fungi, and 29 to 46 (NucleoSpin) and 38 to 78 (DNeasy)μg/μl when isolating DNA of dried fungi (Table 2). In comparison, during isolation with the CTAB method, 36 to 131 μg/μl of total DNA was obtained from cultured fungi and 97 to 110 μg/μl of total DNA in the case of dried fungi. The newly adapted silicon dioxide method provided 6 to 56 and 35 to 80 μg/μl of total DNA respectively (Table 2). In general, the A260/A280 ratio of isolated DNA was in the range 1.4–1.6 in the case of isolation using commercial kits, 1.7–1.9 using the CTAB method, or in the range 1.2–1.9 for the silica capture method (Table 2). Analysis of the total DNA isolated from fungi using commercial kits for isolation tested by electrophoresis on agarose gel showed the best results and highest integrity of the DNA. However, in the case of CTAB and silica methods, we also obtained good-quality DNA in most cases (Table 2).

**Fig. 1:**
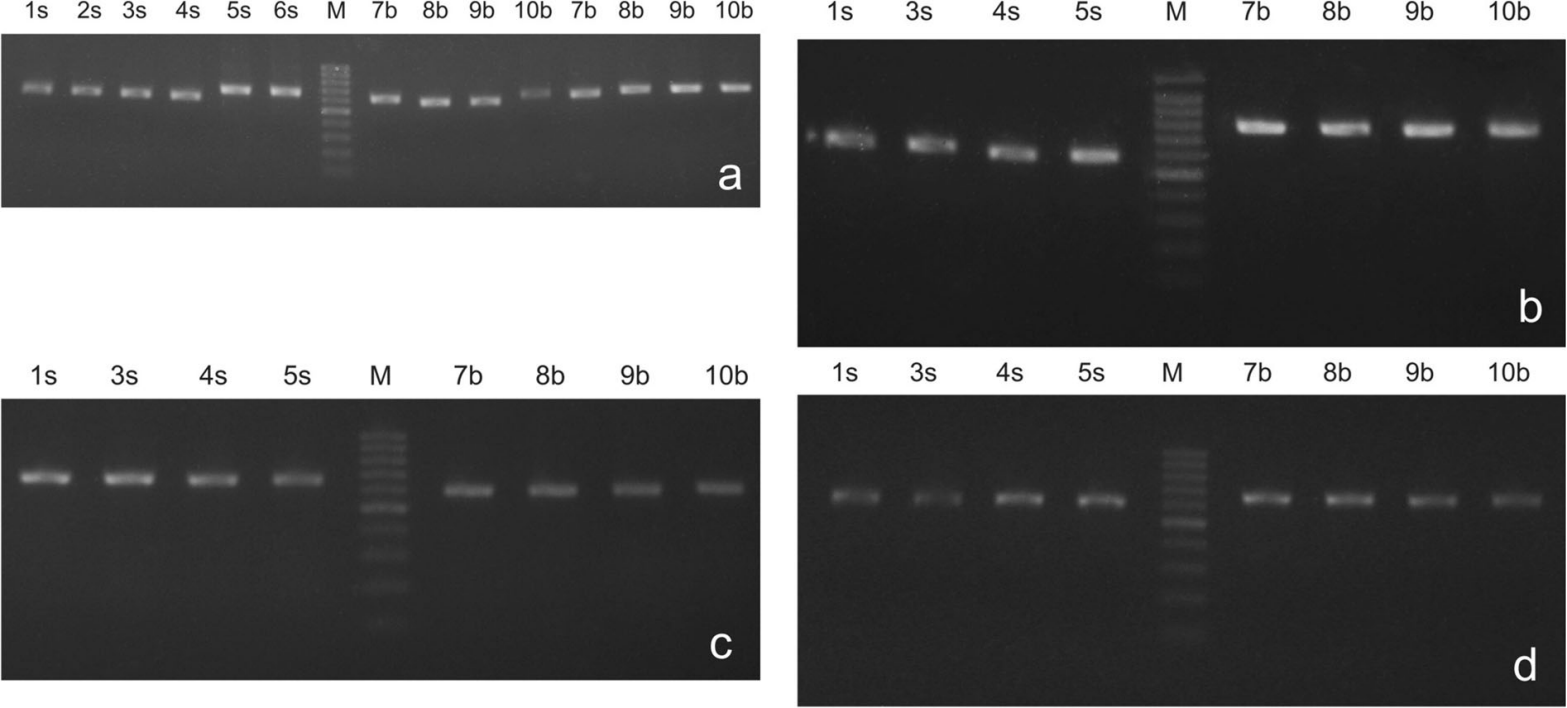
**a** DNA isolate evaluation - silica dioxide powder, PCR amplification of the ITS region. For sample details, see Table 2. Lane M, 100 bp DNA ladder. Samples 7b–10b were amplificated twice; **b** DNA isolate evaluation -NucleoSpin® Plant II/Macherey Nagel Kit, PCR amplification of the ITS region. For sample details, see Table 2. Lane M, 100 bp DNA ladder; **c** DNA isolate evaluation -DNeasy Plant Mini Kit/Qiagen, PCR amplification of the ITS region. For sample details, see Table 2. Lane M, 100 bp DNA ladder; **d** DNA isolate evaluation-CTAB method, PCR amplification of the ITS region. For sample details, see Table 2. Lane M, 100 bp DNA ladder.

**Fig. 2:**
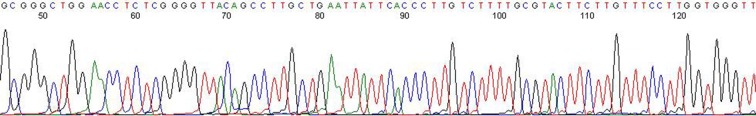
DNA isolate evaluation - silica dioxide method (this study.) An example of sequencing of amplified ITS region.

We also performed an operational test for DNA quality, performing amplification of one of the most often used DNA regions in fungi: the internal transcribed spacers (ITS1 and ITS2) that refer to the spacer DNA situated between 18S and 5.8S rRNA genes and in between 5.8S and 26S, respectively. As expected, in the case of all tested methods, we obtained very good results. A specific PCR amplification product was observed in the gel for all types of isolation methods (Fig. 1a–d). In addition, some PCR products were sequenced (Fig. 2).

## Discussion

All methods, including the silicon dioxide method, produced good-quality DNA usable for future usage in molecular research. The method based on silicon dioxide particles was developed for routine isolation of nucleic acids *(*Boom et al. 1990), later adapted for research on viruses (Malinowski 1997) and improved for detection of a viral pathogen in potato tissue extract (Zacharzewska et al. 2014). But, as far as we know, this paper is the first one to show its application to isolation, amplification, and sequencing of fungal genes: both from macroscopic tissue and fungi cultured on agar slants, known to be a recalcitrant substrate for DNA isolation, and later in species determination. We also provide a comparison of all used techniques, including commercially available DNA isolation kits. In general, the DNA obtained with all of the tested methods was of high quality, allowing for future experiments, such as amplification or cloning experiments. Between tested kits, both the NucleoSpin Plant II and DNeasy Plant Mini Kit were equally effective. Using both, we obtained DNA of high quality, however, in lower concentration than in the CTAB and silica particles methods. The procedure of using commercial kits was much shorter than that of the CTAB method, however longer than that of using silica powder (Table 2). Longer isolation time in the case of commercial kits can be compensated by a much higher quality of the DNA obtained compared with other methods. Better quality allows for a wider range of possible applications, e.g., AFLP which requires good quality of the DNA. Using the silicon dioxide method allowed for much faster isolation (Table 2), which may compensate for the lower quality of DNA. The method yielded enough DNA to be used for PCR amplification and sequencing. Efficiency and sensitivity of the PCR reaction from both diluted and undiluted isolates obtained with the silica method were the same as that of PCR amplification where isolates prepared with commercial kits or CTAB isolation procedure served as the DNA matrix. The DNA sequencing results, shown as clear individual nucleotide peaks, are clearly distinguishable for all tested samples (Fig. 2).

In general, the silicon dioxide protocol tested during this study offers a number of advantages over other DNA isolation procedures. One of them is its low cost. The silicon dioxide powder is a cheap chemical, non-toxic, and easy to use in the lab. The general cost per isolation was estimated to be 0.6 EUR; however, it could be lower (down to 0.3 EUR) if bulk amounts of silica were to be bought. It is over six times cheaper than the cost of the CTAB method and up to 10–11 times cheaper than the cost of commercial kits with a spin column. The used working solution of the silicon dioxide matrix can be prepared in a short time from the stock solution, being ready in several minutes. This allows not only for saving time but also for fast isolation when needed. Depending on the volume of silica powder used, this method allows for efficient elution in small volumes (5–10 μl), which may be useful when only small amounts of the studied tissue are available, or only the fungal spores are studied. This would not be possible in the case of isolation using commercial kits, where a minimum of 30–50 μl of used elution volume is required; changing of which can influence the total amount of isolated DNA. In addition, the silica-based method we present does not need any specialized equipment, e.g., expensive ultracentrifuges, needed in the case of isolating DNA using the CsCl gradient.

In summary, it can be stated that using silica powder (silicon dioxide matrix-based) for capturing DNA is effective, fast, easy to use, and cheaper than using commercial kits. In addition, as was shown, the method can be adapted for fast and efficient isolation of PCR amplification products directly from the PCR tube or from the bands cut from the gel. One of the advantages is that no specialized equipment, additional time-consuming centrifugation, or yield-reducing precipitation steps (as in the CTAB method) are required for efficient DNA isolation. All of the tested and presented modifications of the method, serving possible changes to the general product quality, were described in the paper. It should allow researchers using the described approach easily to modify and optimize the whole procedure of isolation and fit into specific conditions. We hope that the described method will be helpful in environmental monitoring and allow for fast and easy determination of species.

## Abbreviations

AP1 buffer: lysis buffer (DNeasy® Plant Mini Kit, Qiagen, Hilden, Germany)
L2, L6 buffer: buffers prepared according to Boom et al. (1990) with some modifications proposed by Zacharzewska et al. (2014)
PL1: CTAB-based lysis buffer (NucleoSpin® Plant II DNA Kit, Macherey Nagel, Düren, Germany)
PL2: sodium dodecylsulfate lysis buffer (NucleoSpin® Plant II DNA Kit, Macherey Nagel, Düren, Germany)
rcf: relative centrifugal force (G-force)
SDS: sodium dodecylsulfate
TE: Tris-EDTA buffer solution
